# Techniques for Percutaneous Transesophageal Gastro-tubing in the Management of Gastric Leak or Dysphasia After Sleeve Gastrectomy

**DOI:** 10.1007/s11695-022-05909-0

**Published:** 2022-01-20

**Authors:** Takashi Oshiro, Taiki Nabekura, Tomoaki Kitahara, Ayako Takenouchi, Yuki Moriyama, Natsumi Kitahara, Makoto Nagashima, Shinichi Okazumi

**Affiliations:** Department of Surgery, Toho University Sakura Medical Center, 564-1 Shimoshizu, Sakura, Chiba 285-8741 Japan

**Keywords:** Percutaneous transesophageal gastro-tubing (PTEG), Double PTEG, Sleeve gastrectomy, Obesity

## Introduction

Sleeve gastrectomy (SG) has become the most widely performed bariatric and metabolic surgery worldwide [1] owing to its technical simplicity and favorable weight loss effects. However, a tube-shaped stomach after sleeve gastrectomy could be a negative factor that retards healing of leakage [2] and hinders the creation of percutaneous endoscopic gastrostomy.

Although the nasogastric tube (NGT) is a useful tool for managing sleeve leakage and feeding enteral nutrition, it is not appropriate for long-term placement because of poor patient tolerability and potential complications [3].

Percutaneous transesophageal gastro-tubing (PTEG) is a viable alternative to nasogastric decompression or enteral access in patients who require long-term tube placement [4]. Furthermore, one additional indwelling tube through the same opening of the primary PTEG (double PTEG) simultaneously enables enteral nutrition access and gastric drainage separately [5].

## Materials and Methods

We experienced 4 (1 male, 3 female) cases of PTEG in patients after SG (3 for early sleeve leakage and 1 for dysphagia due to amyotrophic lateral sclerosis (ALS): case 3). PTEG for sleeve leakage was performed following the failure of endoscopic treatments including clipping and stenting. One patient underwent double PTEG due to refractory sleeve leakage (case 4). The first and third cases were performed under local anesthesia and intravenous sedation, while the two other cases were subjected to general anesthesia for postural stability.

The PTEG kit was supplied by SB-Kawasumi Laboratories, Inc. (Tokyo, Japan). The procedure was performed as previously described by Oishi et al. [6]. Briefly, the procedure was performed as follows. A tube with rupture free balloon (RFB) was inserted into the upper esophagus through a nostril. The RFB was prepared using chloroethylene to prevent its rupture by needle puncture and to increase its ultrasonographic visibility. Inflated RFB with mixed contrast medium and saline was punctured percutaneously using an introducer needle through the left side of the neck under ultrasonography and fluoroscopy. A matching guidewire was passed through the needle into the RFB, and then threaded into the esophageal lumen following RFB tube removal. After dilating the path around the guidewire using a strong and flexible dilator, the indwelling tube was advanced over the guidewire. An additional intraluminal tube was inserted through the same opening of the primary PTEG using a second guidewire and a dilator with a peel-away sheath. The double PTEG technique is shown in Video S1 (case 4).

## Results

At the time of PTEG, the body mass index (BMI) of each of the four patients was 35 kg/m^2^, 40 kg/m^2^, 30 kg/m^2^, and 43 kg/m^2^, respectively, and BMI loss from pre-SG was 9 kg/m^2^, 19 kg/m^2^, 14 kg/m^2^, and 8 kg/m^2^, respectively. PTEG was successful in all patients without any adverse events and enabled tube feeding the following day. The tube was removed without any complications in the cases of sleeve leakage. Two of them were healed by the timing of the tube removal on post-PTEG day 96 for case 1 and 43 for case 2. Although intractable sleeve leakage (case 4) required revision surgery for laparoscopic total gastrectomy 3 months after double PTEG, long-term tube feeding did not necessitate parenteral nutrition, and another intraluminal drainage tube with intermittent suction could contribute to infection control. The orifice of the tubing at the left neck was completely closed within a few days after tube removal. Patients with ALS received enteral nutrition support using PTEG for over a year without any complications.

## Conclusion

PTEG (or double PTEG) may be considered as a potential therapeutic option to overcome the difficulties resulting from a sleeve-like stomach after SG. PTEG could be an alternative to NGT for long-term placement and minimize the burden on patients requiring long-term tube placement. Appropriate patient selection for balanced benefit from PTEG is challenging in obese patients needing bariatric and metabolic surgery.

## Supplementary Information

Below is the link to the electronic supplementary material.

Supplementary file1 (MP4 342067 kb)
